# Characterization of Patients Seeking Care at a Sexual Health Clinic Who Report Engaging in Exchange Sex

**DOI:** 10.1097/OLQ.0000000000001666

**Published:** 2022-07-04

**Authors:** Medhavi Bole, Christine M. Khosropour, Sara N. Glick, Lindley A. Barbee, Matthew R. Golden, Shireesha Dhanireddy, Julia C. Dombrowski

**Affiliations:** From the Departments of ∗ Medicine; † Epidemiology, University of Washington; ‡ Public Health—Seattle and King County HIV/STD Program, Seattle, WA

## Abstract

People who exchange sex (PWES) for money or drugs are at increased risk for poor health outcomes and may be reluctant to engage in health services.

**Methods:**

We conducted a cross-sectional analysis of patients seen for new problem visits at the Public Health—Seattle and King County Sexual Health Clinic between October 2010 and March 2020 who reported exchanging sex for drugs or money in a computer assisted self-interview. We analyzed demographics; sexually transmitted infections (STIs), human immunodeficiency virus (HIV), and hepatitis C virus (HCV) history; and HIV preexposure prophylaxis (PrEP) use, stratified by gender. We compared characteristics of people who ever versus never exchanged sex using χ^2^ tests and analyzed the visit reason and outcomes among PWES.

**Results:**

Among 30,327 patients, 1611 (5%) reported ever exchanging sex: 981 (61%) cisgender men, 545 (34%) cisgender women, and 85 (5%) transgender and gender diverse persons. Compared with people who never exchanged sex, PWES were more likely to report homelessness (29% vs 7%, *P* < 0.001), injection drug use (39% vs 4%, *P* < 0.001), prior STIs (36% vs 19%, *P* < 0.001), prior HIV diagnosis (13% vs 5%, *P* < 0.001), and prior HCV diagnosis (13% vs 2%, *P* < 0.001). People who exchange sex came to the clinic seeking STI tests (60%), HIV tests (45%), and care for STI symptoms (38%). Overall, 320 (20%) PWES were diagnosed with STIs, 15 (1%) were newly diagnosed with HIV, and 12 (1%) initiated PrEP at the visit.

**Conclusion:**

People who exchange sex have complex barriers to care, and sexual health clinic visits present an opportunity to improve health services for this population.

## Abstract 2

Five percent of sexual health clinic patients reported exchanging sex for drugs or money. This population faces complex barriers to care and needs improved services.

People who exchange sex (PWES) for money, drugs, or other resources are vulnerable to several poor health outcomes, including human immunodeficiency virus (HIV) and sexually transmitted infection (STIs).^1–4^ In the United States, HIV prevalence among women who exchange sex is estimated to be approximately 17% (ranging from 0.3% to 32% in different studies),^5^ and at least 8.8 times higher than HIV prevalence among women in the general population.^6^ Among men who exchange sex, the estimated HIV prevalence is 19.3%.^7^ Cooccurring health conditions such as substance use disorders, and social and structural factors such as poverty, unstable housing, stigma, and criminalization of sex work increase health risks and create barriers to accessing health services.^5–11^ Improving sexual health services, and health care services more broadly, for PWES is an urgent need.^6,11–13^

For many PWES, the use of primary health care is limited and to the extent they seek medical care, it is often in an emergency department setting. Among women who exchange sex and inject drugs surveyed in the 2016 Seattle area National HIV Behavioral Surveillance survey, 55% reported that they did not get the medical care they needed in the past year.^14^ Common reasons for avoiding care include negative experiences in the health care system, need for walk-in services and evening or weekend hours, concerns about cost and transportation, as well lack of awareness of services.^14–16^ When PWES do access health care, they often do not disclose to medical providers that they exchange sex.^9,14–16^

The literature is limited regarding effective models of care for engaging PWES in the US health care system. Two published examples include a peer-based multiservice clinic in San Francisco and a continuity clinic model colocated with a community-based organization that serves people living homeless in Seattle.^9,17^ These clinics address the specific health care needs of PWES and decrease emergency department utilization for outpatient services.^18^ Sexually transmitted infection specialty clinics (sexual health clinics) are 1 venue in which PWES may seek care, particularly for urgent problems, such as STI symptoms. These visits may provide an opportunity to diagnose infections (STIs, HIV, hepatitis), provide linkage to treatment, and engage patients in preventive care such as HIV preexposure prophylaxis (PrEP) and long-acting reversible contraception. However, little is known about the extent to which PWES seek care in sexual health clinics, characteristics of PWES seen in these clinics, and the opportunity for improved care delivery in this setting. Furthermore, the existing research in this area focuses primarily on cisgender women, and less is known about cisgender men and transgender people who exchange sex.

The purpose of this study was to characterize patients in the Public Health—Seattle and King County (PHSKC) Sexual Health Clinic who report engaging in exchange sex and identify opportunities for improved services.

## METHODS

### Study Design and Population

We conducted a descriptive, cross-sectional analysis of patients seen for new problem visits at the PHSKC Sexual Health Clinic from October 6, 2010, to March 20, 2020, in which patients reported a history of exchanging sex for money or drugs. All patients seen for a new problem visit were asked to complete the standard clinical computer-assisted self-interview (CASI), which included information on demographics, sex and drug related behaviors, relevant health history, and reason for the visit. Patients who did not speak English were not asked to complete the CASI, and patients could opt out of the CASI, or they may decline to answer questions or skip questions in the CASI. The analytic population included all patients who were seen for new problem visits, completed a CASI, and answered questions on sex assigned at birth, gender identity, and whether they had ever received money or drugs in exchange for having sex. Individual patients were the unit of analysis, and for patients with multiple visits, data from the most recent visit were analyzed. Patients who reported exchanging sex at 1 or more visits but not at all clinic visits were categorized as having ever exchanged sex, and data from the clinic visit in which they most recently reported exchanging sex was analyzed.

### Data Collection and Measures

We defined PWES as patients with a lifetime history of exchanging sex, as indicated by answering “yes” to the question, “Have you ever received money or drugs in exchange for having sex with someone?” A follow-up question about the recency of exchanging sex changed during the follow up period. Those who completed the survey before December 19, 2018, and indicated “yes” to the lifetime history question were then asked to report the date of last exchange sex. Patients who completed the survey after December 19, 2018, were asked, “In the last 12 months, have you received money or drugs for having sex with someone?” We defined people has having exchanged sex if they answered yes to this question or reported the date of last exchange sex as within 1 year before their visit.

Patients were defined as cisgender if the current gender identity and sex assigned at birth were the same. Transgender patients were defined as having either current transgender identity or current gender identity different from their sex assigned at birth. Before May 3, 2016, sex assigned at birth was collected during clinic registration, and gender identity was assessed using a 1-step question that asked, “Do you identify as male, female, or transgender?” that allowed 4 response options: male, female, transgender male to female, and transgender female to male. A 2-step gender identity question was implemented on May 3, 2016, that asked, “What gender do you consider yourself?” that allowed for 2 additional response options to the 1-step question: nonbinary/genderqueer, and a write-in option.^19^ The second question asked the sex assigned at birth, “What sex was recorded on your original birth certificate?” For the analysis, we included transgender patients and those who identified as genderqueer, nonbinary, or other identities into a single category of transgender and gender diverse (TGD) persons.

Age, race, ethnicity, and housing status were collected during clinic registration. The following measures were collected by self-report in the CASI: gender of sex partners, sexual orientation, number of male partners in the past 2 and 12 months, injection drug use (IDU) (ever and past year) and type of drugs injected, history of STIs in the past year, previous diagnosis of HIV, previous diagnosis of hepatitis C virus (HCV), HIV and HCV treatment history, PrEP use (ever and current), and reason for coming to the sexual health clinic. The wording of some of the CASI questions varied over time, and some data were collected for only subsets of the analysis period (Table, Supplemental Digital Content 1, http://links.lww.com/OLQ/A841, lists the CASI questions and date range). Other questions were only posed to some patients based on gender and gender of sex partners. For example, the number of male partners in the past 2 months was collected for cisgender men and cisgender women, and the number of male partners in the past 12 months was collected for cisgender men and TGD persons. Sexually transmitted infection history was analyzed from May 2012 onward because of incomplete data before that time.

The assessment of PrEP use in PHSKC Sexual Health Clinic began in June 2013. Patients who were cisgender men or TGD persons who have sex with men were asked, “Have you ever taken HIV medicines to prevent yourself from getting HIV?” and in December 2018, assessment of current versus past PrEP use began. Cisgender women were not queried about PrEP use because men who have sex with men (MSM) and transgender were the priority focus for PrEP uptake since the majority of people diagnosed with HIV in King County and Washington State are MSM.^20^

During the period of analysis, PHSKC Sexual Health Clinic policy for HIV testing was to test at least once a year, STI screening per Centers of Disease Control and Prevention guidelines were adapted based on anatomy and exposure, and HCV testing for persons who inject drugs.

### Statistical Analyses

Before the analysis, we compared 2 definitions of PWES: those who reported a lifetime history of exchange sex (5%) versus exchange sex in the past 12 months (2%) (Figure, Supplemental Digital Content 2, http://links.lww.com/OLQ/A841, is a flowchart of patients who exchanged sex ever by gender. Figure, Supplemental Digital Content 3, http://links.lww.com/OLQ/A841, is a flowchart of patients who exchanged sex in the past 12 months by gender. Table, Supplemental Digital Content 4, http://links.lww.com/OLQ/A841, compares the characteristics of patients by exchange sex status in the past 12 months). These populations were not meaningfully different, and all analyses in this study defined PWES as those with a lifetime history of exchange sex. Patients who reported exchanging sex at some visits but not others were categorized as PWES. We used descriptive analyses to evaluate demographics; behavioral data; STIs; HIV and HCV history; PrEP use, and reason for the visit. For variables collected during only part of the analysis period, we calculated percentages using the denominator of patient encounters completed after the question was added to the CASI. We calculated the proportion of PWES based on demographics, behavioral data, STIs, HIV and HCV history, and PrEP use. We compared the characteristics of PWES versus patients who reported never exchanging sex using 2-sided χ^2^ tests with a statistical significance level of 0.05.

We compared the characteristics of PWES stratified by gender (cisgender men, cisgender women, and TGD persons). The rationale was that the context of exchange sex likely differed between these groups.

Among all PWES, we examined the reason for a visit to the sexual health clinic and key visit outcomes selected a priori: new STI diagnosis, HIV and HCV testing and results among those without a prior diagnosis of HIV or HCV, and PrEP initiation among HIV-negative patients not on PrEP. All data were analyzed using Stata version 16.0 (College Station, TX). The University of Washington Human Subjects Division deemed this deidentified analysis exempt from institutional review board.

## RESULTS

### Study Population

During the study period, 30,327 unique patients attending 64,680 clinic visits had a completed CASI with an answer to the exchange sex question. Overall, 1611 (5%) reported ever exchanging sex, and these patients attended a total of 3097 visits.

### Characteristics of PWES

Compared with people who never exchanged sex, PWES were more likely to be cisgender women (34% vs 24%; *P* < 0.001) or TGD persons (5% vs 1%; *P* < 0.001) (Table 1). More TGD persons reported ever exchanging sex (21%) than compared with cisgender women (7%) and cisgender men (4%). Among 9201 cisgender MSM, 580 (6%) reported ever exchanging sex (data not shown). The age and race distributions varied slightly between the populations. Substantially more PWES reported homelessness or unstable housing (29% vs 7%, *P* < 0.001) and IDU (39% vs 4%, *P* < 0.001).

**TABLE 1:** Characteristics of Patients Attending Public Health—Seattle and King County Sexual Health Clinic for a New Problem by Exchange Sex Status, 2010–2020

	Exchanged Sex Ever, 1611 (5%)	Never Exchanged Sex, 28,716 (95%)		
Characteristics	n (%)	n (%)	Row % That Exchanged Sex Ever	*P*
Age, yr				
≤18	37 (2)	503 (2)	7	
19–24	292 (18)	4892 (17)	6	0.005
25–29	339 (21)	6699 (23)	5
30–49	778 (48)	13,095 (46)	6
≥50	165 (10)	3527 (12)	4
Race				
Asian/Pacific Islander	52 (3)	2748 (10)	2	
Black	324 (20)	4842 (17)	6		<0.001
Mixed/multiracial	80 (5)	733 (3)	10		
Native American/Alaskan Native	32 (2)	317 (1)	9		
Unknown/missing/refused	110 (7)	2680 (9)	4		
White	1013 (63)	17,396 (61)	6		
Hispanic ethnicity	148 (9)	2569 (9)	5	0.742	
Gender			
Cisgender man	981 (61)	21,542 (75)	4	<0.001
Cisgender woman	545 (34)	6850 (24)	7	
TGD person	85 (5)	324 (1)	21	
Homelessness*				
Homeless/unstable housing	256 (29)	980 (7)	21	<0.001
Stable housing	619 (71)	13,569 (93)	4	
Ever injected drugs (IDU)				
Yes	630 (39)	1140 (4)	36	<0.001
No	967 (60)	27,526 (96)	3	
Missing	14 (1)	50 (0)	22	
Bacterial STIs^†^ and Trichomonas in the past 12 mo^‡^				
Yes	499 (36)	4628 (19)	10	<0.001
No	844 (61)	18,692 (77)	4	
Missing	37 (3)	443 (2)	8	
Prior HIV diagnosis				
Yes	208 (13)	1376 (5)	13	<0.001
No	1157 (72)	20,693 (72)	5	
Unknown/missing	246 (15)	6647 (23)	4	
Last viral load undetectable, among those with HIV				
Yes	139 (67)	998 (73)	12	0.040
No	38 (18)	165 (12)	19	
Missing/unknown	31 (15	213 (15)	13	
Prior HCV diagnosis				
Yes	212 (13)	538 (2)	28	<0.001
No	1116 (69)	21,843 (76)	5	
Unknown/missing	283 (18)	6335 (22)	4	
HCV+ received treatment				
Yes	50 (24)	201 (37)	20	<0.001
No	72 (34)	126 (23)	36	
Unknown/missing	90 (42)	211 (39)	30	
Currently on PrEP^§¶^				
Yes	79 (33)	1041 (34)	7	0.638
No	97 (40)	1274 (42)	7	
Missing	64 (27)	729 (24)	8	

ART, antiretroviral therapy.

*Homelessness data from July 1, 2015, onward.

^†^Bacterial STI includes (gonorrhea, chlamydia, and syphilis).

^‡^Data from May 21, 2012, onward.

^§^Among those who tested negative for HIV.

^¶^Data only from December 19, 2018, onward.

Compared with people who never exchanged sex, PWES were more likely to have had an STI diagnosis in the past 12 months (36% vs 19%, *P* < 0.001), a prior HIV diagnosis (13% vs 5%, *P* < 0.001), or a prior HCV diagnosis (13% vs 2%, *P* < 0.001). However, PWES were proportionately less likely to self-report having an undetectable HIV viral load (67% vs 73% of those with HIV, *P* = 0.04) or past HCV treatment (24% vs 34% of those with HCV, *P* < 0.001). Among 240 PWES without a prior HIV diagnosis December 2018 onward, 33% reported currently being on PrEP, similar to the proportion among those who never exchanged sex.

### PWES Stratified by Gender

Among PWES, 61% were cisgender men, 34% were cisgender women, and 5% were TGD persons (Table 2). Of the 981 cisgender men, 82% reported sex with other men. Compared with cisgender men and TGD persons, cisgender women were more likely to identify as black (26%), multiracial (7%), or Native American/Alaskan Native (4%) and were more likely to report homelessness (41%).

**TABLE 2:** Characteristics of Public Health—Seattle and King County Sexual Health Clinic Patients Who Reported Exchange Sex, Stratified by Gender, 2010–2020 (N = 1611)

Characteristic	Cisgender		TGD Persons
	Men	Women	n (%)
	n (%)	n (%)	85 (5)
	981 (61)	545 (34)	
Age, yr			
≤18	19 (2)	15 (3)	3 (4)
19–24	165 (17)	114 (21)	13 (15)
25–29	206 (21)	102 (19)	31 (36)
30–49	477 (49)	263 (48)	38 (45)
≥50	114 (12)	51 (9)	0
Race			
Asian/Pacific Islander	36 (4)	13 (2)	3 (4)
Black	175 (18)	139 (26)	10 (12)
Mixed/multiracial	36 (4)	39 (7)	5 (6)
Native American/Alaskan Native	11 (1)	21 (4)	0
Unknown/missing/refused	73 (7)	28 (5)	9 (11)
White	650 (66)	305 (56)	58 (68)
Hispanic ethnicity	99 (10)	38 (7)	11 (13)
Homelessness*			
Homeless/unstable housing	141 (25)	96 (41)	19 (22)
Not homeless	414 (75)	139 (49)	66 (78)
Gender of sex partners			
Any male	808 (82)	525 (96)	80 (94)
Any female	339 (35)	168 (31)	33 (39)
Any gender diverse	81 (8)	13 (2)	49 (58)
None	13 (1)	7 (1)	1 (1)
Missing	5 (1)	6 (1)	0
IDU, ever			
Yes	392 (40)	210 (39)	28 (33)
No	581 (59)	330 (61)	56 (66)
Missing	8 (1)	5 (1)	1 (1)
Drugs injected among those with IDU			
Heroin	144 (37)	142 (68)	10 (36)
Cocaine	102 (26)	80 (38)	3 (11)
Methamphetamine	309 (79)	131 (62)	19 (68)
Other	76 (19)	46 (220	5 (18)
Bacterial STIs^†^ and Trichomonas in the past 12 mo^‡^			
Yes	365 (42)	100 (23)	34 (40)
No	477 (55)	319 (74)	48 (57)
Missing	19 (2)	15 (3)	3 (4)
STIs in the past 12 mo^‡^			
Gonorrhea	232 (64)	40 (40)	24 (71)
Chlamydia	228 (62)	58 (58)	23 (68)
Syphilis	141 (39)	11 (11)	7 (21)
Trichomonas	7 (2)	31 (31)	4 (12)
Prior HIV diagnosis			
Yes	195 (20)	4 (1)	9 (11)
No	663 (68)	429 (79)	65 (76)
Unknown/missing	123 (13)	112 (21)	11 (13)
Last viral load undetectable, among those with HIV			
Yes	132 (68)	2 (50)	5 (56)
No	35 (18)	1 (25)	2 (22)
Missing/unknown	28 (14)	1 (25)	2 (22)
Prior HCV diagnosis			
Yes	118 (12)	90 (17)	4 (5)
No	707 (72)	331 (61)	78 (82)
Unknown/missing	156 (16)	124 (23)	3 (4)
HCV+ received treatment			
Yes	28 (24)	22 (24)	0
No	33 (28)	38 (42)	1 (25)
Unknown/missing	57 (48)	30 (33)	3 (75)
Currently on PrEP^§,¶^			
Yes	68 (48)	NA	11 (32)
No	75 (52)		22 (65)
Missing	0		1 (3)

*Homelessness data from July 1, 2015, onward.

^†^Bacterial STI includes (gonorrhea, chlamydia, and syphilis).

^‡^Data from May 21, 2012, onward.

^§^Among those who tested negative for HIV, MSM and TGD persons only.

^¶^Data only from December 19, 2018, onward.

Injection drug use was reported by 40% of cisgender men, 39% of cisgender women, and 33% of TGD persons. Although methamphetamine was the most common IDU among cisgender men (79%) and TGD persons (68%), heroin was the most common substance injected by cisgender women (68%).

Having an STI in the past 12 months was more frequently reported by cisgender men (42%) and TGD persons (40%), than cisgender women (23%). Although gonorrhea was the most frequent STI diagnosis reported by cisgender men (64%) and TGD persons (71%), chlamydia was the most frequent STI reported by cisgender women (58%).

Prior HIV diagnosis was more common among cisgender men (20%) than TGD persons (11%) and cisgender women (1%). Of the 195 cisgender men with a prior HIV diagnosis, 132 (68%) had an undetectable viral load. Cisgender women were more likely to have a prior HCV diagnosis (17%) but less likely to have had HCV treatment (42%). Among 143 cisgender men not previously diagnosed with HIV, 68 (48%) reported currently being on PrEP, and among 34 TGD persons negative for HIV, 11 (32%) reported currently being on PrEP.

### Reason for Visit and Visit Outcomes

**Figure 1:**
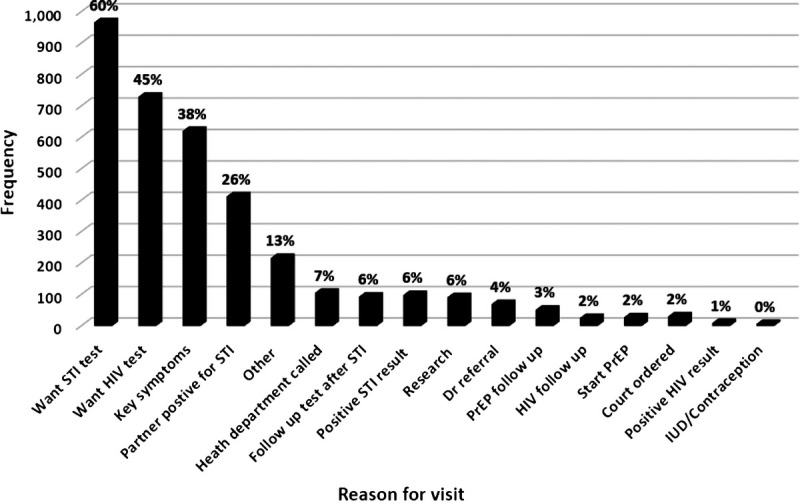
Reason for a visit to Public Health—Seattle and King County Sexual Health Clinic among those who reported exchanged sex ever, 2010–2020. Dr indicates doctor; IUD, intrauterine device.

The most common reasons reported for visiting the PHSKC Sexual Health Clinic among PWES was wanting STI testing (60%), followed by seeking HIV testing (45%), evaluation of STI symptoms (38%), and having a partner diagnosed with an STI (26%) (Fig. 1). Of the 1611 PWES, 320 (20%) were diagnosed with 1 or more STI at the clinic visit included in this analysis; 11% gonorrhea, 6% chlamydia, and 5% syphilis (Table 3). Among PWES, 1026 (64%) were tested for HIV at the clinic visit (15 [1%] tested positive) and 300 (19%) were tested for HCV (73 [5%] tested positive). Among 97 PWES without prior diagnosis of HIV who reported not being on PrEP, 12 (1%) PWES were prescribed PrEP.

**TABLE 3:** Visit Outcomes of People Who Exchange Sex Seen in the Public Health—Seattle and King County Sexual Health Clinic, 2010–2020 (N = 1611)

Visit Outcome	n (%)
STI diagnosis	
Gonorrhea	173 (11)
Chlamydia	103 (6)
Syphilis	73 (5)
HIV screening	
Negative	1011 (63)
New positive	15 (1)
Previous positive	223 (14)
Not tested at visit	362 (23)
HCV screening	
Negative	224 (14)
Positive	73 (5)
Missing sample/unsatisfactory	3 (0)
Not tested at visit	1311 (81)
Initiated PrEP	
Yes	12 (1)
No/missing	1599 (99)

## DISCUSSION

In this study, 5% of patients in the PHSKC sexual health clinic reported a history of exchange sex, half of whom were cisgender MSM. Homeless or unstable housing and IDU were common among PWES, especially cisgender women. Although PWES were more likely to report a prior HIV or HCV diagnosis compared with people who never exchanged sex, they were less likely to report HIV viral suppression or HCV treatment. About half of cisgender MSM who reported exchange sex were on PrEP and very few started PrEP at the clinic visit. Similar to the overall population of patients in the sexual health clinic, PWES sought care predominantly for STI/HIV testing and evaluation of STI symptoms.

To our knowledge, only a few prior studies have focused on PWES seen in sexual health clinics. One from Rhode Island also found higher prevalence of IDU, STIs, HIV, and HCV among PWES compared with the general sexual health clinic patient population.^21^ The prevalence of HIV among patients in our analysis was comparable to that study for cisgender men (12% vs 10%) but higher for cisgender women (10% vs 3%). Our finding that half of the PWES in the sexual health clinic were cisgender MSM, and that a relatively small number of cisgender women PWES were seen over the 11-year period suggests that our clinic may not be effectively reaching cisgender women who exchange sex. The majority of patients in sexual health clinics in many US cities are cisgender men.^22,23^ Sexual health clinics are preferred because of convenience, cost, confidentiality, and expert care.^24,25^ Women who inject drugs and exchange sex surveyed in the 2016 Seattle area NHBS survey reported receiving STI testing in private and community clinics more frequently than in sexual health clinics.^14^ In contrast, a prior study in Colorado Springs estimated that 80% of all “female prostitutes” were seen in the health department STD clinic during 1970 to 1990. One factor that likely contributed to this was health department outreach efforts, including “recruiting efforts … where and when prostitutes work.”^26^ Models of care, such as community clinics colocated with other services or peer-based clinics, may have more success engaging this population.^9,17^

These results add to prior studies, suggesting that PWES are at higher risk for poor HIV and HCV outcomes. In our study, 67% of PWES reported HIV viral suppression compared with 73% in those who never exchanged sex. A national surveillance study in 2009 to 2014 also found 67% viral suppression among people living with HIV who exchanged sex compared with 76% of those who did not exchange sex.^27^ People who exchange sex have been a priority population for initiating PrEP in Washington state.^28^ Although we found levels of PrEP use among cisgender men and TGD persons who exchange sex in this analysis (48% and 32%, respectively) to be higher than prior studies of PrEP uptake (range, 5%–22%),^21,29^ PrEP is still underutilized among PWES who are MSM or TGD persons, women who exchange sex for money or drugs, and persons who inject drugs. The barriers to HIV and HCV treatment and to PrEP in this population are likely similar to barriers to health care in general. Our finding of considerable overlap between homelessness and IDU, particularly in cisgender women, confirms similar findings in other settings^1,8,30^^–34^ and indicates the complex life circumstances that can make it difficult for PWES to engage in treatment or adhere to daily medications.

Our findings have relevance for services in sexual health clinics more broadly. The first step in improving health services for PWES is asking about exchange sex. This is not routinely done in all settings, and although screening likely under ascertains exchange sex, tailoring care requires identification of the population. Sexual health clinics (including our own) can improve HIV and STI screening and connection to PrEP for PWES. Ideally, sexual health clinics could also provide services to link PWES to HIV and HCV treatment. Partnering with PWES to improve programs and conduct research in the future will likely improve the effectiveness and quality of our programs and ensure that the research addresses needs identified by the community.

Our study had several strengths. First, we looked at CASI data for PWES over an 11-year period, which captured a relatively large population of PWES. To our knowledge, this study is the first to evaluate differences among PWES by gender in a sexual health clinic setting. However, our analysis was limited to 1 sexual health clinic and has unknown generalizability to other settings or for populations of PWES who do not seek sexual health clinic services. The question used in the CASI to ascertain exchange sex focused on “money or drugs,” whereas the context of exchange sex may also include other resources and survival needs, which may be different for cisgender women compared with cisgender MSM. The prevalence of exchange sex is almost certainly underreported to the CASI. Finally, our focus on a lifetime history of exchange sex may have missed significant differences among people who are currently exchanging sex compared with those who have not recently exchanged sex.

In conclusion, our study shows that although exchange sex is uncommonly reported among sexual health clinic patients, patients who present for services and self-identify as PWES likely have complex medical and social needs. Sexual health clinic visits present an opportunity to engage PWES in health services and improve HIV and HCV outcomes.

## Appendix

For further references, please see “Supplemental References,” http://links.lww.com/OLQ/A842.
